# Upregulation of NLRP3 Inflammasome in the Tears and Ocular Surface of Dry Eye Patients

**DOI:** 10.1371/journal.pone.0126277

**Published:** 2015-05-11

**Authors:** Liangliang Niu, Shujie Zhang, Jihong Wu, Ling Chen, Yan Wang

**Affiliations:** 1 Department of Ophthalmology, Eye and ENT Hospital of Fudan University, Shanghai, China; 2 Experimental Research Center, Eye and ENT Hospital of Fudan University, Shanghai, China; 3 Department of Ophthalmology, University of Hong Kong, Hong Kong, China; Queen Mary University of London, UNITED KINGDOM

## Abstract

**Purpose:**

To evaluate the mRNA and protein expressions of NLRP3 inflammasome and its downstream inflammatory factors in human dry eye.

**Methods:**

We recruited 54 patients with Sjögren’s syndrome dry eye (SSDE), 50 patients with non-Sjögren’s syndrome dry eye (NSSDE), and 46 healthy controls. Tear film breakup time (TBUT), Schirmer I test, and fluorescein staining (FL) were performed on all subjects. Tear samples were obtained to analyze the inflammatory cytokine levels of IL-1β and IL-18 via enzyme-linked immunosorbent (ELISA). Conjunctival impression cytology (CIC) specimens were collected to detect the mRNA expression of NLRP3, caspase-1, IL-1β, and IL-18 using quantitative RT-PCR, and the protein expression of NLRP3 and caspase-1 by Western blotting.

**Results:**

NLRP3 mRNA expression showed higher levels in both dry eye groups compared with controls, with a comparably significant elevation in the SSDE group (relative 2.47-fold upregulation, *p*<0.05). NLRP3 protein expression was also increased in SSDE group (relative1.94-fold upregulation) compared with the controls. mRNA expression of caspase-1 was significantly upregulated in both SSDE (relative 1.44-fold upregulation, *p*<0.05) and NSSDE (relative 1.32-fold upregulation, *p*<0.05). Procaspase-1 protein level was increased in SSDE (relative 1.84-fold upregulation) and NSSDE (relative 1.12-fold upregulation) versus controls; and caspase-1 protein expression was also increased in SSDE (relative 1.49-fold upregulation) and NSSDE (relative 1.17-fold upregulation) compared with the controls. The patients with SSDE and NSSDE had higher IL-1β and IL-18 mRNA values and protein expressions than the controls did. The relative mRNA expression of IL-1β upregulated 3.59-fold (*p*<0.001) in SSDE and 2.13-fold (*p*<0.01) in NSSDE compared with the controls. IL-1β protein level also showed significant upregulation in SSDE (*p*=0.01; vs. controls groups). IL-18 mRNA expression levels were significantly upregulated in the SSDE (relative 2.97-fold upregulation, *p*=0.001) and NSSDE (relative 2.05-fold upregulation, *p*=0.001) groups compared with the controls; tear IL-18 concentrations were also significantly increased in the SSDE (*p*<0.001) and NSSDE (*p*<0.05) groups.

**Conclusions:**

In the current study, we found that mRNA and protein expressions of NLRP3 inflammasome were upregulated in human dry eyes, especially in SSDE; the downstream inflammatory factors caspase-1, IL-1β, and IL-18 were also elevated in dry eye patients. These observations suggest the involvement of NLRP3 inflammasome in the onset and development of the inflammation in dry eye.

## INTRODUCTION

Dry eye is one of the most common ocular surface disorders that significantly affect the quality of human life. Although the pathogenesis of dry eye has not been established clearly, there is increasing evidence that immune-based inflammation on the ocular surface might play a prominent role in the pathological damage of dry eye [1, 2]. Numerous studies have shown increased levels of inflammatory cytokines and apoptotic modulators in the tear and conjunctival epithelium of dry eye patients and animal models, including interleukin (IL)-1α, IL-1β, IL-8, IL-6,tumor necrosis factor (TNF)-α, interferon (IFN)-α, intercellular adhesion molecule (ICAM)-1, human leukocyte antigen (HLA)-DR, cluster of differentiation (CD)-40, CD-40L, Fas, chemokine receptors CCR5 and CXCR3[3–10]. However, the precise mechanism of the onset and development of inflammation in dry eye remains unclear.

Inflammasome plays a key role in inflammation and innate immunity. The inflammasome is an intracellular protein complex that is stimulated by pathogen-associated molecular patterns (PAMPs) or danger-associated molecular patterns (DAMPs). It can activate procaspase-1, causing cleavage of pro-IL-1β and pro-IL-18. Bioactive cytokines then initiate or amplify diverse downstream signaling pathways and drive proinflammatory processes [11–13]. Among different types of inflammasomes, the Nod-like receptor family pyrin domain containing 3 (NLRP3) inflammasome is currently one of the most well-characterized subtypes. Previous studies have demonstrated critical roles for NLRP3 inflammasome activation in the immune responses of many diseases, such as asthma [14], T-cell-dependent immune complex glomerulonephritis[15], acute graft-versus-host disease (GvHD)[16], systemic lupus erythematosus (SLE)[17], metabolic disorders like type 2 diabetes[18], gout, and pseudogout [19]. Recently several reports also found NLRP3 inflammasome involved the pathology of the age-related macular degeneration [20,21,22].

However, limited information is available on the association of NLRP3 inflammasome with dry eye. A recent study reported that reactive oxygen species could trigger NLRP3 inflammasome and lead to caspase-1 auto-activation and maturation of proinflammatory cytokines IL-1β in dry eye murine models [23]. However, the alteration of NLRP3 inflammasome in the tears and ocular surface of dry eye patients has not been reported until now. In the current study, we prospectively recruited patients with SSDE and NSSDE. Tear samples and conjunctival impression cytology specimens were collected to evaluate the mRNA and protein expressions of NLRP3 inflammasome and its downstream inflammatory factors-caspase-1, IL-1β and IL-18. The results were compared with healthy control subjects.

## MATERIALS AND METHODS

### Patients

Fifty-four SSDE subjects (six males, 48 females; mean age, 54.07±9.69 years) and fifty NSSDE subjects (eight males, 42 females; mean age, 48.51±11.42 years) were recruited for this prospective study and examined at the dry eye subspeciality clinic of the Eye and ENT Hospital of Fudan University. Forty-six age- and sex-matched healthy subjects were included as controls (nine males, 37 females; mean age, 48.24±13.89 years). Both eyes of all subjects were included in this study. All procedures adhered to the Declaration of Helsinki, and ethics approval was obtained from Ethics Committee of Eye and ENT Hospital of Fudan University, Shanghai, China. Written consent was obtained from all patients. All participants underwent a clinical evaluation visit to determine entry eligibility before a second visit, in which ocular samples were collected.

Dry eye disease was diagnosed according to diagnostic criteria reported previously [24]. Briefly, patients with (1) dry eye-related symptoms, (2) positive staining with fluorescein, or (3) Schirmer I test results ≤5 mm or a tear film breakup time (TBUT) value <5 seconds were diagnosed as having definite dry eye. Diagnosis of SS was confirmed with the cooperation of an internist, according to the American—European Consensus Criteria (2002) [25]. All dry eye patients had used only artificial tears and topical anti-inflammatory drugs such as cyclosporine A; patients using steroids were excluded. Subjects were excluded if they wore contact lenses, had active ocular disease, or had recent eye surgery, including punctal plugs or cautery.

### Tear Film Breakup Time (TBUT) and Cornea Fluorescein Staining (FL)

TBUT and FL were measured using prepackaged, sterile fluorescein paper strips (Jingming New Technological Development Co. Ltd, Tianjin, China). First, the fluorescein strip was wetted with 20μl saline, and then the lower tarsal conjunctiva was gently touched with the end of the strip. The patient was asked to blink a couple of times for a few seconds to assure adequate mixing of the dye. The time from the last complete blink to the appearance of the first corneal black spot in the stained-tear film was examined three times, and the mean value of the measurements was calculated. A TBUT value of less than five seconds was considered to be abnormal. After measuring the TBUT, the cornea FL score was evaluated. The FL score of the cornea ranged from 0 to 9 points; any score above 3 points was regarded as abnormal [26].

### Schirmer I Test

The Schirmer I test was performed using prepackaged, sterile paper strips without anesthesia (Jingming New Technological Development Co. Ltd). The rounded bulb end of the strip was folded and placed in the lateral canthus that away from the cornea and left in place for five minutes. After wetting for five minutes, readings were repeated in millimeters.

### Tear Sample Collection and Analysis

In order to analyze inflammatory cytokine levels, tear samples were collected according to the method described previously [27]. To collect the tear samples, 30μl of phosphate-buffered saline were instilled into the inferior fornix without topical anesthetics. After a gentle blink, tear samples were taken from each eye using a 20μl capillary tube. All of the tear samples were gained from the lateral canthus, that parallel to the ocular surface, without stimulating reflex tearing, followed by immediate transfer to a 0.5ml Eppendorf tube and centrifugation at 1000 rpm for three minutes at 4°C. The supernatants then were reserved at -80°C. The amounts of tear IL-18 and IL-1β were measured using an enzyme-linked immunosorbent assay (ELISA) kit (eBioscience, San Diego, CA) according to the manufacturer’s instructions. The optical density of each well was determined at a wavelength of 450 nm. Samples were considered positive when the signal was higher than the background signal (modified Krebs solution) and was within the range of the standard curve. The experiment was repeated twice.

### Conjunctival Impression Cytology (CIC) Samples Collection

Conjunctival impression cytology (CIC) samples were collected according to the method described previously [28,29]. The CIC specimens were obtained after administration of topical anesthesia with 0.4% oxybuprocaine. Two separate sterile membrane filters (0.45*μ*m; Millipore, Boston, MA) that were soaked in distilled water for a few hours and dried at room temperature were applied to adjacent superior and inferior temporal bulbar conjunctiva, pressed gently by a glass rod, and then removed. The filter paper collected from the left eye was transferred immediately to a 1.5ml Eppendorf tube containing 1ml of RNA stabilization reagent (Qiagen, Germantown, Germany) for mRNA expression measurement. A CIC sample from the right eye was placed in an empty 1.5ml Eppendorf tube for western blotting to evaluate the protein expression. All samples were immediately placed on ice until transferred to −80°C for storage.

### RNA Isolation from CIC Samples and Reverse Transcription

Tubes containing 1ml of RNA stabilization reagent and CIC samples were allowed to thaw at room temperature and then were vortexed for 2 min. Extraction of total RNA proceeded according to the manufacturer’s protocol (RNeasy Mini Kit; Qiagen, Valencia, CA). The final isolation step was conducted with 35μl of RNase-free water. RNA concentration was measured by NanoDrop 2000 (Thermo Scientific, Wilmington, DE) prior to reverse transcription. The procedure of cDNA synthesis from RNA samples was performed with a thermal cycler (Thermal Technology, Santa Rosa, CA) using Oligo dT Primer and Random 6 mers according to the manufacturer’s instruction (PrimeScript RT reagent Kit; Takara, Osaka, Japan).

### Quantitative RT-PCR of the CIC Samples

NLRP3, caspase-1, IL-1β, and IL-18 mRNA expression of CIC samples were detected using a SYBR Green real-time polymerase chain reaction (PCR) kit according to the manufacturer’s instructions (SYBR Premix Ex Taq; Takara, Osaka, Japan). *β-actin* was used as an endogenous control gene for this analysis. Sequence data for gene amplification in quantitative PCR (qPCR) is summarized in Table 1. Real-time qPCR was performed with a ViiA 7 Real-Time PCR System (Life Technologies, Pleasanton, CA). Collected data was analyzed and fold-expression changes were calculated using the comparative CT method (2^-ΔΔCT^) of relative quantification with ViiA 7 Software (Life Technologies).

**Table 1:** Sequence Data for Gene Amplification in Quantitative RT-PCR.

Gene	Forward Primer	Reverse Primer
NLRP3	5’-TCCTCGGTACTCAGCACTAATCAG-3’	5’-GGTCGCCCAGGTCATTGTTG-3’
Caspase-1	5’-AAGACCCGAGCTTTGATTGACTC-3’	5’-AAATCTCTGCCGACTTTTGTTTCC-3’
IL-1β	5’-TATTACAGTGGCAATGAGG-3’	5’-ATGAAGGGAAAGAAGGTG-3’
IL-18	5’-ATAGCCAGCCTAGAGGTA-3’	5’-ATCAGGAGGATTCATTTC-3’
β-actin	5’-CCCTGGACTTCGAGCAAGAG -3'	5’-TCACACTTCATGATGGAGTTG-3’

### Western Blotting of the CIC Samples

Expression of the NLRP3 and caspase-1 proteins of the CIC samples were determined by western blot analysis. CIC samples from 6 subjects were put together and lysed with radioimmunoprecipitation assay buffer (1% Triton X-100, 1% deoxycholate, 0.1% SDS) on ice for one hour. The lysates were centrifuged at 12,000 rpm at 4°C for 10 min to obtain the supernatant. Each 60μl cell sample was added, with protein loading buffer, and degenerated in a heating block for 5 min, after which the proteins were separated by 12% sodium dodecyl sulfate-polyacrylamide gel electrophoresis and transferred onto a polyvinylidene fluoride membrane (0.45 *μ*m; Millipore, Bedford, MA). The membrane was blocked in 2% bovine serum albumin (BSA) for one hour at room temperature and incubated with the specific primary antibodies (diluted 1:1000) overnight at 4°C. After washing with phosphate buffer solution containing Tween-20 five times, the membranes were probed with horseradish peroxidase-conjugated secondary antibodies (diluted 1:5000 by 2% BSA). In accordance with conventional methods, β-actin level was measured at the same time as the internal control. The results were quantified by analysis for grayscale using Gel-Pro Analyzer software (Media Cybernetics, Rockville, MD). The following primary antibodies were used: anti-β-actin, anti-NLRP3 and anti-caspase-1 (Abcam, Boston, MA).

### Statistical Analysis

SPSS 19.0 (IBM Corporation, Armonk, NY) was used to evaluate significance, and GraphPad Prism 6 (GraphPad Software Inc., La Jolla, CA) was used to generate figures and tables. The Kruskal—Wallis test was used for comparisons involving tear film BUT, Schirmer I test values, ocular vital staining scores, protein and mRNA expression levels of NLRP3, caspase-1, IL-1β and IL-18. Data were shown as mean ±standard deviation(SD). *P* <0.05 was considered significant.

## RESULTS

### Demographic Characteristics and Clinical Examination Parameters

A total of 150 subjects were enrolled in this study, including 54 SSDE patients, 50 NSSDE patients, and 46 healthy sex- and age-matched controls. The baseline ocular surface and tear function parameters of the three groups are presented in Table 2. Significant differences were found in each parameter among the three groups (*p*<0.05).

**Table 2:** Summary of Diagnostic Tests for Study Groups.

Group	TBUT(s)	FL (Points)	Schirmer I(mm/5min)
SSDE	2.59±1.00^a^ ^,^ ^b^	4.48±2.31 ^a^ ^,^ ^b^	3.04±2.56^a^ ^,^ ^b^
NSSDE	3.30±0.92^a^	1.43±0.82 ^a^	5.16±4.51^a^
Control	10.00 ±3.00	0.02±0.15	11.5±6.21

Abbreviations: SSDE = Sjögren's syndrome dry eye; NSSDE = non-Sjögren's syndrome dry eye; TBUT = tear break-up time; FS = fluorescein score;

^a^ *p <0*.*05*, compared with normal controls, Kruskal-Wallis test.

^b^ *p<0*.*05*, compared with NSSDE patients, Kruskal-Wallis test.

### NLRP3 mRNA and Protein Expressions in Dry Eye

**Fig 1:**
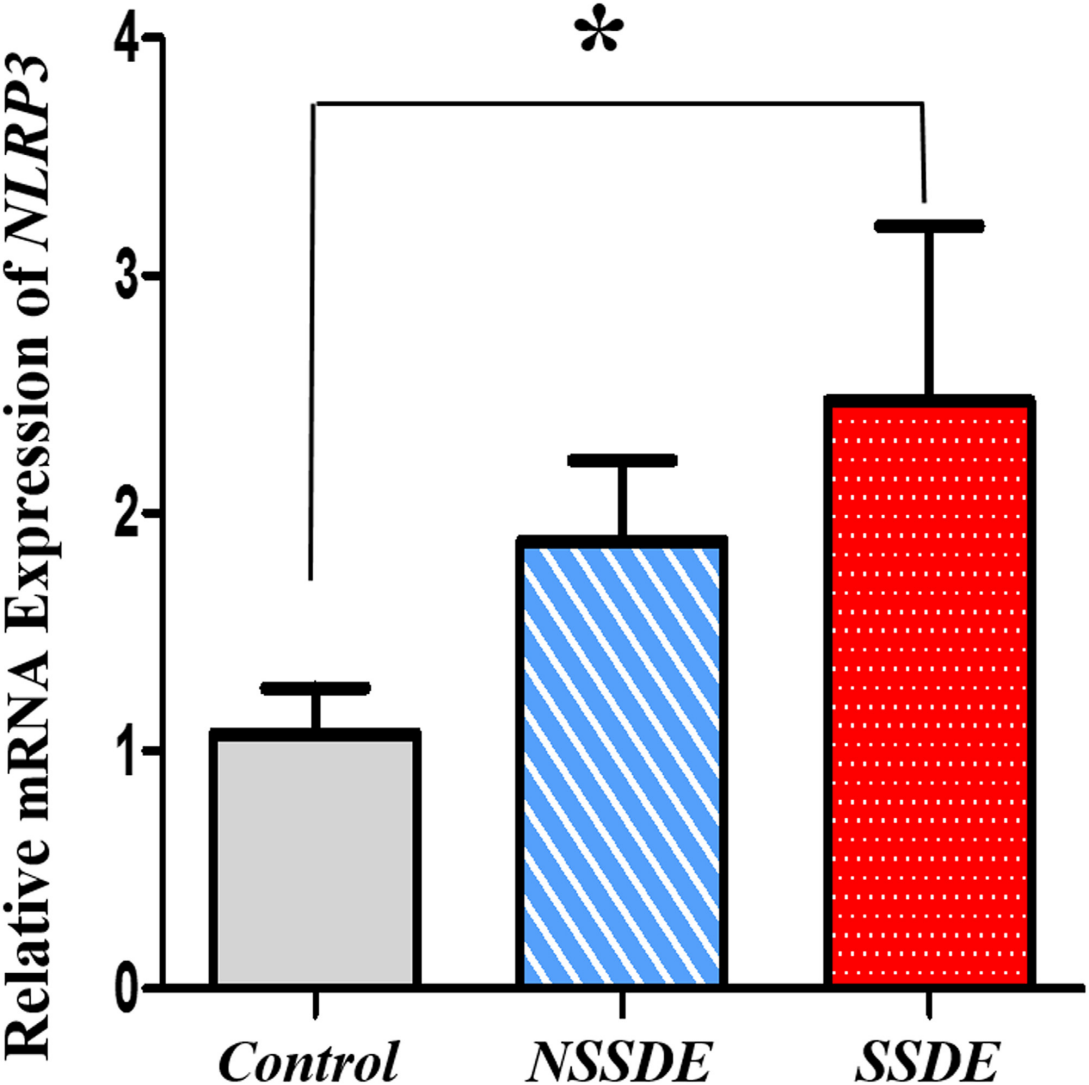
Upregulation of NLRP3 mRNA expression in the CIC samples of dry eye patients. Quantitative analysis of mRNA expression of NLRP3 showed significantly upregulation in SSDE. (*p<0.05, compared with the controls, Kruskal-Wallis test).

Since NLRP3 inflammasome has a vital function in modulating inflammation and immune responses [11–13, 14–19], we detected its expression in human dry eye. With the use of real-time RT-PCR, the relative expression of NLRP3 mRNA showed higher levels in both the SSDE group (relative 2.47-fold upregulation) and the NSSDE group (relative 1.88-fold upregulation) compared with the controls, with a comparably significant elevation in the SSDE group (*p*<0.05, Fig 1).

**Fig 2:**
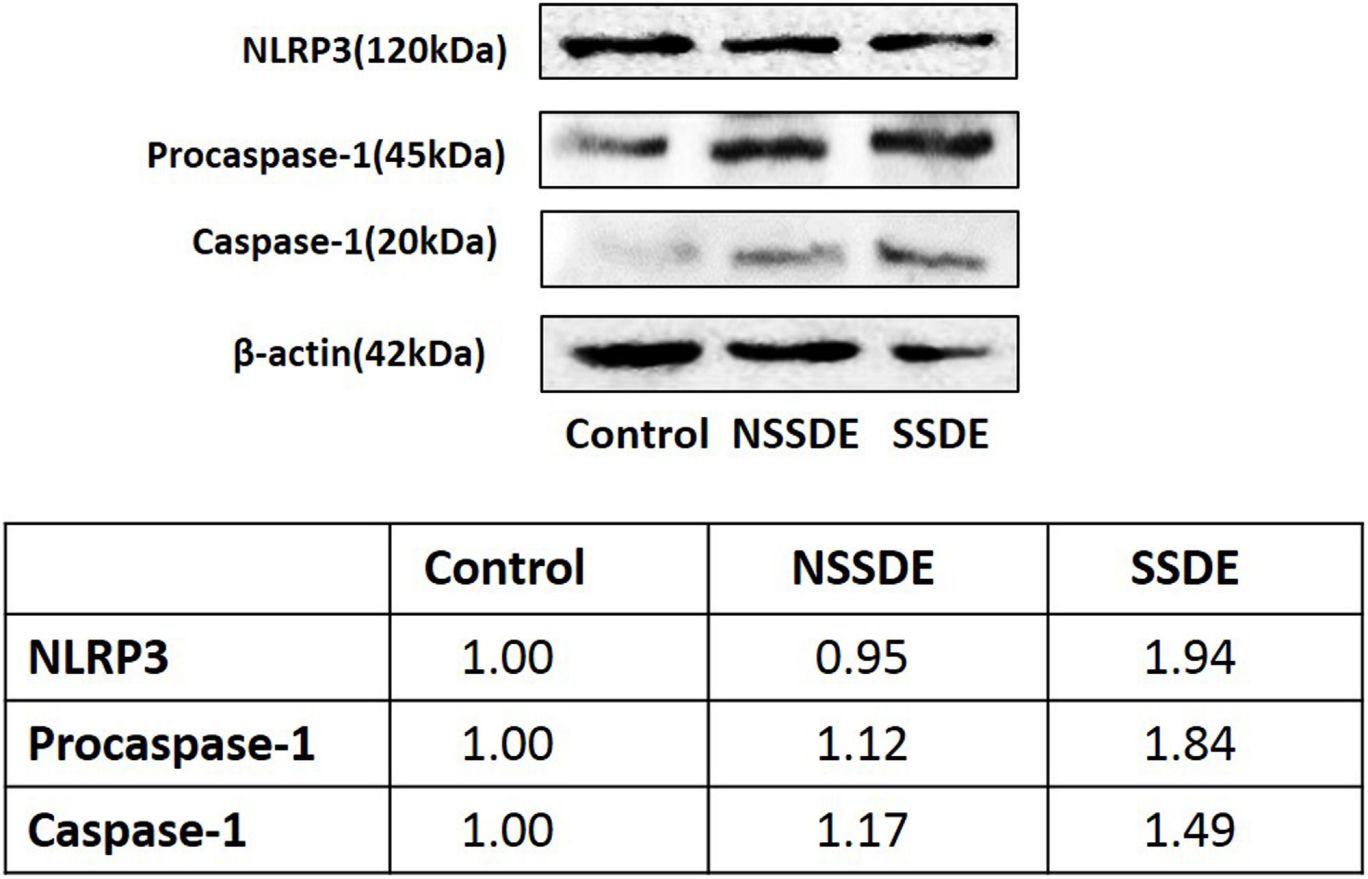
Western blot and densitometric analyses of NLRP3, procaspase-1,and caspase-1 protein expressions in the eyes of controls, NSSDE, and SSDE subjects. Expression of 120-kDa NLRP3 increased in SSDE versus controls. Expression of 45-kDa procaspase-1 and 20-kDa caspase-1 showed increased protein expressions in SSDE and NSSDE compared with controls.

NLRP3 protein level was detected by western blot analysis. The densitometric analyses showed that NLRP3 protein expression was clearly increased in the SSDE group (relative 1.94-fold upregulation) compared with the healthy control subjects, but no significant differences were found in NLRP3 protein expression between the NSSDE and control subjects (Fig 2).

### Caspase-1 mRNA and Protein Expressions in Dry Eye

**Fig 3:**
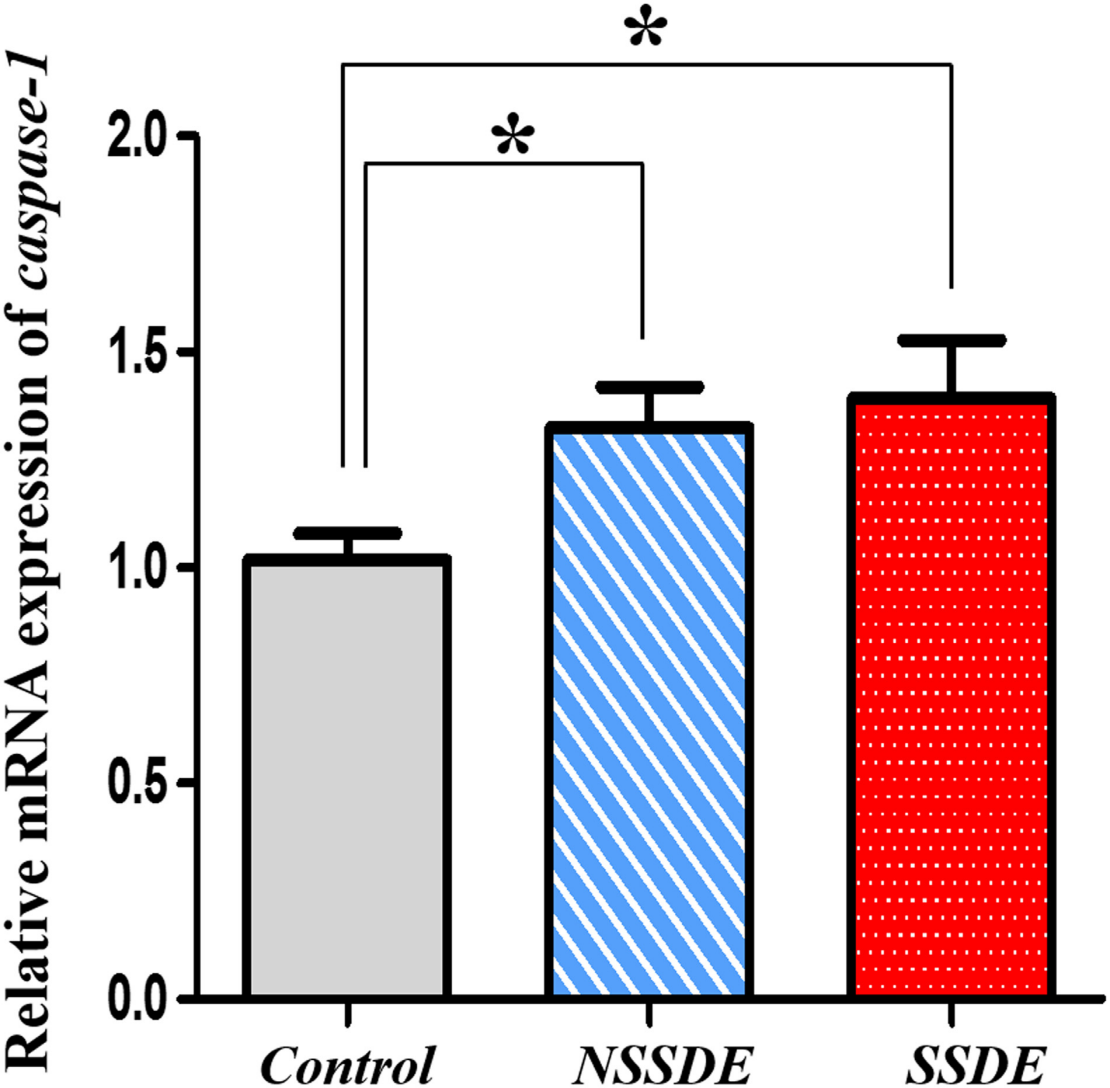
Upregulation of caspase-1 mRNA expression in the CIC samples of dry eyepatients. Quantitative PCR results of caspase-1 demonstrated significantly upregulation in SSDE and NSSDE groups. (*p<0.05, versus the controls, Kruskal-Wallis test).

Inflammasome is a kind of multiprotein complex, after stimulated by the outside signals, it can lead to the activation of caspase-1[11]. Therefore, we further detected the expression of caspase-1. The qPCR results of caspase-1 mRNA expression showed significant upregulation in both the SSDE group (relative 1.44-fold upregulation) and the NSSDE group (relative 1.32-fold upregulation) compared with the control eyes (*p*<0.05, Fig 3).

Western blot and densitometric analyses of procaspase-1 (pro-enzyme) and caspase-1 (active form) are shown in Fig 2. An increase of the procaspase-1 protein level was found in both SSDE and NSSDE groups in comparison with the control eyes, with 1.84-fold and 1.12-fold levels, respectively. The SSDE and NSSDE groups also had 1.49-fold and 1.17-fold greater caspase-1 expressions than the control eyes.

## IL-1β mRNA and Protein Expressions in Dry Eye

**Fig 4:**
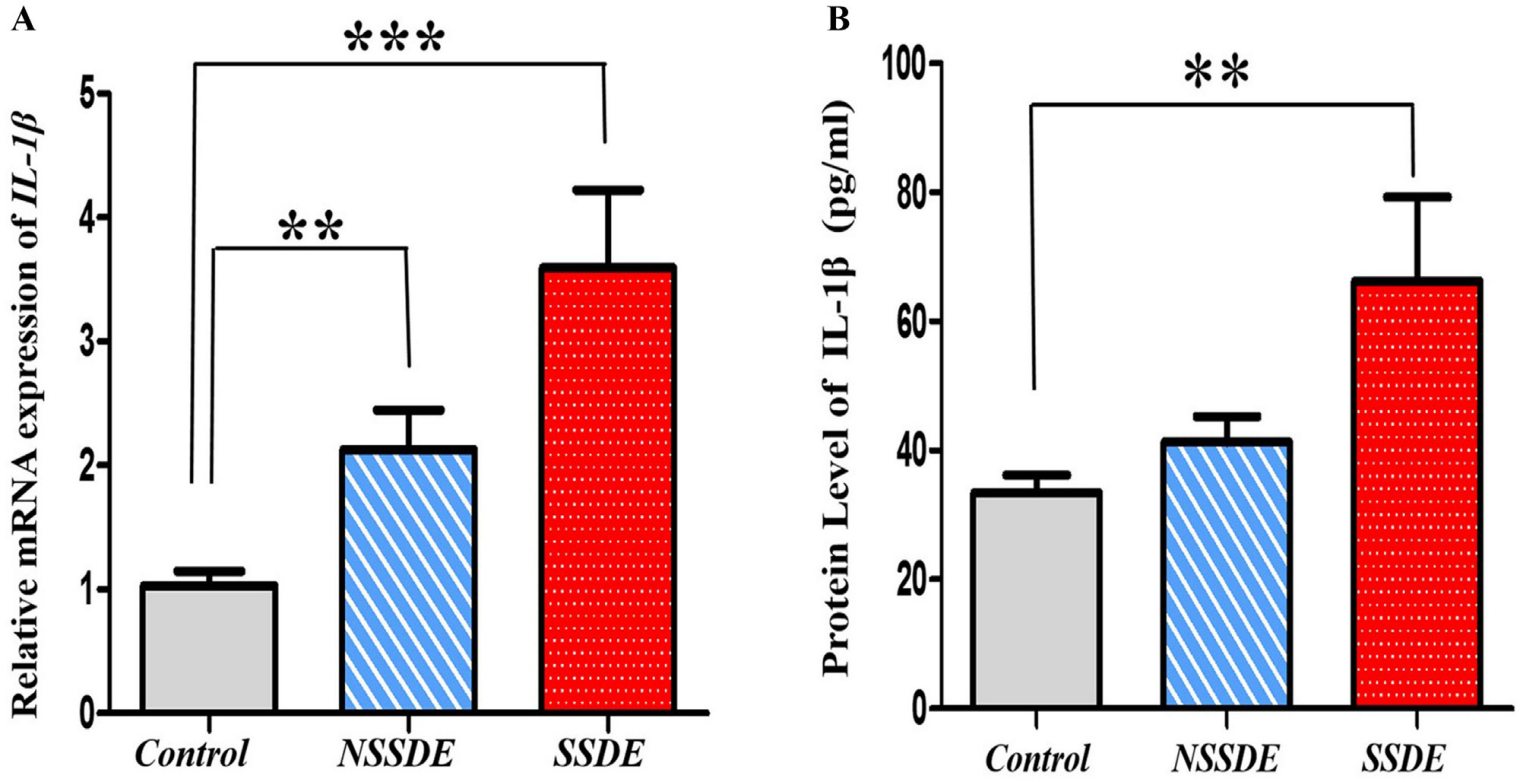
Upregulation of IL-1β expression in the tear and CIC samples of dry eye patients. (**A**) Quantitative RT-PCR for IL-1β mRNA expression in CIC specimens showed significant upregulation in eyes with SSDE and NSSDE (***p≤0.001, **p≤0.01, compared with the controls, Kruskal-Wallis test). (**B**) Tear IL-1β protein expressions via ELISA demonstrated higher levels in SSDE group compared with controls. (**p≤0.01, compared with the controls, Kruskal-Wallis test).

Since the activation of caspase-1 can be processed in regulation the secretion of IL-1β and IL-18[30], we next examined the inflammatory cytokines IL-1β and IL-18. Quantitative PCR results of IL-1β mRNA expression demonstrated significant upregulation in the SSDE eyes (3.59-fold relative upregulation, *p*<0.001) and NSSDE eyes (2.13-fold relative upregulation, *p*<0.01) compared with the control eyes (Fig 4A).

Tear IL-1β levels were measured by ELISA. The mean protein levels of IL-1β in tears were 33.42±11.25 pg/mL in the control subjects, 41.33±20.34 pg/mL in patients with NSSDE, and 66.18±68.13 pg/mL in patients with SSDE. The level of IL-1β increased significantly in the tears of patients with SSDE compared with the control subjects (*p* = 0.01). The NSSDE group also had a higher IL-1β protein expression level compared with the healthy control subjects (Fig 4B).

### IL-18 mRNA and Protein Expressions in Dry Eye

**Fig 5:**
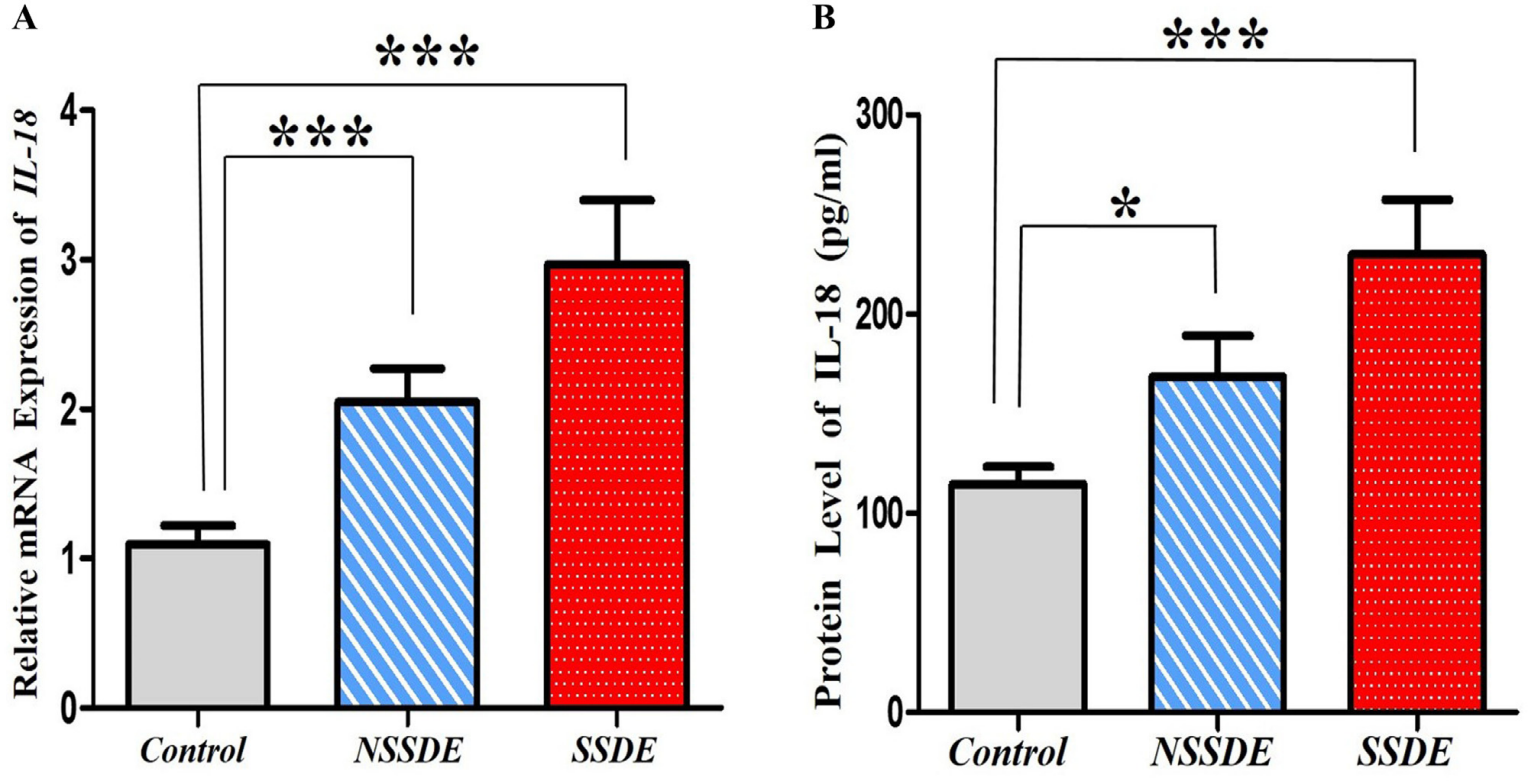
Upregulation of IL-18 expression in the tear and CIC samples of dry eye patients. (**A**) Significant elevation of IL-18 mRNA level was found in SSDE and NSSDE groups using quantitative RT-PCR.(***p≤0.001, compared with the controls, Kruskal-Wallis test). (**B**) ELISA analyses of IL-18 protein expression demonstrated tear IL-18 increased significantly in SSDE and NSSDE groups. (*p<0.05, ***p≤0.001, compared with the controls, Kruskal-Wallis test).

As shown in Fig 5A, IL-18 mRNA expression in eyes with SSDE (2.97-fold relative upregulation) and NSSDE (2.05-fold relative upregulation) was significantly higher compared with the control eyes (*p* = 0.001).

Protein expression of tear IL-18 was detected using ELISA. The mean levels of IL-18 in tears were 114.504±34.341pg/mL in controls, 168.364±78.018 pg/mL in patients with NSSDE, and 229.767±107.010pg/mL in patients with SSDE. A significant increase was observed in the SSDE group (*p*<0.001) and NSSDE group (p<0.05) compared with the control subjects (Fig 5B).

## DISCUSSION

NLRP3 inflammasome plays a vital role in modulating innate or adaptive immune responses. Much valuable work has implicated its importance in the onset and development of many inflammatory related diseases [14–19]. Recently, Baldini et al reported increased expressions of the P2X_7_ receptor-NLRP3 inflammasome, caspase-1 and IL-18 in the salivary gland of Sjögren’s syndrome patients. Those results suggested the involvement of NLRP3 inflammasome-capase-1-IL-18 axis in the development of primary Sjögren’s syndrome exocrinopathy [31]. Immune-based inflammation on the ocular surface has been acknowledged as playing a prominent role in the pathological damage of dry eye [1,2]. However, the exact mechanism of inflammation development and activation of various inflammatory cytokines in dry eye is still unclear. We speculated that NLPR3 inflammasome may be involved in the development of dry eye ocular surface inflammation. To study the potential involvement of the NLRP3 inflammasome in dry eye patients, we prospectively recruited patients with SSDE and NSSDE, and collected tear and impression cytology samples to evaluate mRNA and protein expression of NLRP3 inflammasome and its downstream inflammatory factors-caspase-1, IL-1β, and IL-18.

Our results showed a significant increase of NLRP3 mRNA and protein expressions in SSDE subjects versus the controls. Subjects with NSSDE also display a higher level of NLRP3 mRNA expression, although a statistically significant difference was not found in comparison with the controls. These findings indicate that NLRP3 inflammasome may be associated with the development of dry eye ocular surface inflammation. In our study, the baseline tear film BUT, FL and Schirmer test values all were significantly worse in SSDE group than the data in NSSDE group. We speculate the level of NLRP3 expression in SSDE and NSSDE may be associated with the severity of the dry eye disease. In the study of Baldini et al [31], they found increased expression of NLRP3 in the salivary gland of SS patients but not in the salivary gland of NSSDE patients. Pathological alterations are noted in the salivary gland in SS, but this is not the case in NSSDE. However, the ocular surface is affected in both SSDE and NSSDE. In our findings, we found not only a statistical increase in the NLRP3 expression on the ocular surface of SSDE but also a higher expression level of NLRP3 in NSSDE. These findings also suggest that inflammation levels on the ocular surface vary in different types of dry eye.

As previous studies have confirmed that NLRP3 leads up to the activation of caspase-1 in many inflammatory diseases[11,14–19], we found significant upregulation in the levels of caspase-1 mRNA and proteins in both SSDE and NSSDE subjects compared with the control subjects. These findings were consistent with the NLRP3 inflammasome upregulation that we observed. These findings suggest that increased NLRP3 expression may be activating caspase-1 to develop further inflammation in patients with dry eye.

The best-characterized consequence of the caspase-1 activation of NLRP3 inflammasome is secretion of the pro-inflammatory cytokines IL-1β and IL-18 [30]. Once released, these cytokines initiate an inflammatory cascade that leads to the recruitment of innate immune cells and can regulate the subsequent adaptive immune response [32,33]. The current study confirmed a significant increase in IL-1β and IL-18 mRNA and protein expressions in tears and on the ocular surface of dry eyes. These findings suggested that the increased IL-1β and IL-18 expressions might be regulated by NLRP3 inflammasome.IL-1β has been found to decrease tear production via neuronal and hormonal effects [34]. IL-18 has been reported to potentially cause serious damage in the lacrimal and salivary glands of Sjögren’s syndrome patients [10,35]. But the function of IL-18 in dry eye is still not well understood.

In summary, based on tear samples and conjunctival impression cytology specimens of dry eye patients, we found that the expressions of NLRP3 inflammasome and its downstream inflammatory factors-caspase-1, IL-1β and IL-18 are upregulated in dry eye patients, especially in SSDE. These findings suggest the involvement of NLRP3 inflammasome and its downstream inflammatory factors-caspase-1, IL-1β, and IL-18 in the development of ocular surface inflammation in dry eye. To our knowledge, this is the first study to investigate the changes in NLRP3 inflammasome in the tear and ocular surface of dry eye patients. Inflammasome activation could be triggered by many exogenous and endogenous noninfectious stimuli [36,37]. Risk factors for the development of dry eye such as aging, dry environments and contact lens wear [38] may trigger the expression of inflammasome. Higher levels of inflammasome can in turn activate caspase-1 and the secretion of IL-1β and IL-18, which then induce the inflammatory damage of dry eye. In a recent study, Zheng et al [23] found that reactive oxygen species could trigger NLRP3 inflammasome in a dry eye murine model. Understanding the expression of NLRP3 inflammasome associated with the dry eye pathology may clarify factors involved in the progression of the disease and enhance the development of targeted therapies. Further research should focus on the regulatory mechanism of NLRP3 inflammasome in dry eye inflammation; the function of IL-18 in dry eye; and whether inhibition of inflammasome components can serve as a viable target for therapeutic development in dry eye.
